# An open prospective study of amikacin pharmacokinetics in critically ill patients during treatment with continuous venovenous haemodiafiltration

**DOI:** 10.1186/2050-6511-13-14

**Published:** 2012-11-08

**Authors:** Deirdre M D’Arcy, Eoin Casey, Caitriona M Gowing, Maria B Donnelly, Owen I Corrigan

**Affiliations:** 1 Intensive Care Medicine, Tallaght Hospital, Dublin 24, Ireland; 2 School of Pharmacy and Pharmaceutical Sciences, Trinity College Dublin, Dublin 2, Ireland; 3 Pharmacy Department, Tallaght Hospital, Dublin 24, Ireland

**Keywords:** Amikacin, Pharmacokinetics, Haemodiafiltration

## Abstract

**Background:**

The objectives of the current study were to determine amikacin pharmacokinetics in patients undergoing treatment with continuous venovenous haemodiafiltration (CVVHDF) in an Intensive Care Unit (ICU), and to determine whether peak and trough concentration data could be used to predict pharmacokinetic parameters. An open prospective study was undertaken, comprising five critically ill patients with sepsis requiring CVVHDF.

**Methods:**

Peak and trough plasma concentrations and multiple serum levels in a dosage interval were measured and the latter fitted to both a one- and two-compartment model. Blood and ultrafiltrate samples were collected and assayed for amikacin to calculate the pharmacokinetic parameters; total body clearance (TBC), elimination rate constant (k) and volume of distribution (V_d_). The concentration of amikacin in ultrafiltrate was used to determine the clearance via CVVHDF. CVVHDF was performed at prescribed dialysate rates of 1-2l h^-1^ and ultrafiltration rate of 2l h^-1^. Blood was pumped at 200ml/min using a Gambro blood pump and Hospal AN69HF haemofilter. Amikacin dosing was according to routine clinical practice in the Intensive Care Unit.

**Results:**

The multi serum level study indicated that the one compartment model was adequate to characterize the pharmacokinetics in these patients suggesting that peak and trough plasma level data may be used to estimate individual patient pharmacokinetic parameters and to optimise individual patient dosing during treatment with CVVHDF. CVVHDF resulted in an amikacin k of 0.109+/−0.025 h, t_1/2_ of 6.74 +/− 1.69h, TBC of 3.39+/−0.817 h^-1^, and V_d_ of 31.4 +/− 3.27. The mean clearance due to CVVHDF of 2.86 l h^-1^ is similar to the creatinine clearance of 2.74 +/−0.4 lh^-1^. Amikacin was significantly cleared by CVVHDF, and its half life in patients on CVVHDF was approximately 2–3 times that reported in subjects without renal impairment and not undergoing haemodiafiltration for any reason.

**Conclusions:**

CVVHDF contributes significantly to total clearance of amikacin. The use of pharmacokinetic parameter estimates obtained from two steady state serum-drug concentrations (peak and trough) can be used to guide individualised dosing of critically ill patients treated with CVVHDF. This is considered a useful strategy in this patient cohort, particularly in avoiding the risk of underdosing.

## Background

Aminoglycoside antibiotics are used to treat serious infections caused by gram negative microorganisms in intensive care unit (ICU) patients. They are an increasingly popular choice for both empiric and directed therapy as they are less likely to engender resistance than quinolones [1] and have a lesser incidence of promoting Clostridium difficile infection than other antibiotics [2]. The intersection of aminoglycoside and continuous veno-venous haemodiafiltration (CVVHDF) use is an ever increasing likelihood in the ICU setting. The use of amikacin in particular is increasing as it may offer a more extensive spectrum of cover than gentamicin [3], especially with the advent of Extended Spectrum Beta-Lactamase (ESBL) producing organisms.

Elimination of amikacin is mainly via the renal route. Its dosage regimens must therefore be adjusted in severe renal insufficiency to prevent accumulation of the drug to toxic levels and the associated risk of oto- and nephrotoxicity. However, this drug’s hydrophilicity and low molecular weight also make it likely to be cleared by CVVHDF and consequently pharmacokinetic studies during CVVHDF are required to optimise dosing regimens and obtain therapeutic concentrations.

In general, data relating toxicity with aminoglycoside concentrations refer to trough plasma concentrations [4]. Low serum peak aminoglycoside concentrations are associated with an increased risk of clinical failure [5,6] and the emergence of resistant strains [7]. A marked variability in aminoglycoside pharmacokinetic parameters has been reported in critically ill patients [8,9]. For example an increased volume of distribution for aminoglycoside antibiotics has been found in critically ill patients with sepsis [10] and ascites [11]. Additionally, augmented renal clearance has been described in certain patient cohorts, e.g. burns patients.

There are some studies investigating the effects of CVVH on clearance of amikacin [12-14] but there is little published evidence describing the effect of CVVHDF. CVVH depends predominantly on convection alone while CVVHDF involves a combination of solute clearance by diffusion and convection, and is generally expected to have increased removal efficiency over CVVH [15]. One study has suggested that 40% of an amikacin dose could be removed by CVVHDF based on a study of six renal failure patients [16]. Another study recently published investigated pharmacokinetics of amikacin in patients on CVVHDF suffering from sepsis or septic shock following administration of a 25 mg/kg loading dose [17]. This study illustrated that the half life (~6.5 h) of amikacin in these patients on CVVHDF was much lower than reported for patients with renal impairment not receiving dialysis [18] (> 30 hours in anuric patients during the interhemofiltration period). However it did not determine specifically the contribution of CVVHDF to amikacin clearance. Furthermore, it was suggested that accumulation following high loading dose may be an issue in subsequent doses if the dosage interval is not adequately extended. A further recent case report of two patients demonstrated the value of clearance via CVVHDF when administering high doses of amikacin to patients with sepsis due to panresistant *Pseudomonas aeruginosa*. As this combination was used as a therapeutic option to enable higher dosing, no pharmacokinetic analysis was presented in that study [19]. CVVHDF is the preferred modality of continuous renal replacement therapy in the hospital setting of the current work.

A 1-compartment model is considered adequate to describe amikacin pharmacokinetics in most clinical settings to facilitate aminoglycoside dosage adjustments/calculations [4], once peak concentrations are measured after a short distribution phase e.g. 30 minutes after a 30 minute infusion. In the study by Taccone et al. a 2-compartment model was used for the pharmacokinetics analysis of amikacin plasma concentrations following the high loading dose in patients on CVVHDF [17]. Due to the unknown pharmacokinetic behaviour of amikacin in patients on CVVHDF, it was desirable to establish whether a 1-compartment model rather than a 2-compartment model would still be a suitable model selection in these patients. Collection and analysis of multiple serum concentration data is not routine in the clinical setting, therefore assessment of the suitability of the use of peak and trough data only, to calculate pharmacokinetic parameters is mandated. This type of data would be routine in a standard therapeutic drug monitoring (TDM) scenario.

The practice in the hospital setting of the current study prior to the commencement of this study was for the dosage of aminoglycosides to be modified during CVVHDF, on the basis of the best prescribing recommendations of the time. The levels (peak and trough values) obtained indicated potential underdosing and suggested significant CVVHDF induced clearance.

The deficit of data on amikacin pharmacokinetics during treatment with CVVHDF, and the evident potential for underdosing due to clearance via CVVHDF in these patients prompted this study.

The objectives of the study were:

a) to carry out a prospective study of patients treated with amikacin and CVVHDF, and to determine amikacin pharmacokinetic parameters including an estimate of clearance due to CVVHDF

b) to determine whether a 1-compartment model or a 2-compartment model better fitted multiple serum concentration data over the course of one dosage interval in patients on CVVHDF.

c) to determine whether peak and trough data alone would be adequate to calculate pharmacokinetic parameters and subsequent dose recommendations, as this data is obtained during routine therapeutic drug monitoring (TDM) practice.

## Methods

### Patient Selection

The decision to treat with CVVHDF and amikacin was determined prior to the inclusion of the patients in the study. It was an open, prospective, non-interventional study. Demographic data and clinical characteristics are given in Table 1. Estimates of creatinine clearance (CrCl) were obtained using the method of Jelliffe and Jelliffe [20].

**Table 1:** Summary of patients’ clinical and demographic data

**ID**	**Sex**	**Age**	**Diagnosis**	**Infective diagnosis**	**APACHE II Score**^**1**^	**CrCl**^**2**^	**Duration CVVHDF (days)**
P1	M	57	Cirrhosis of liver, sepsis, ARF	Pseudomonas aeruginosa	39	10	7
P2	F	68	Colonic obstruction, post-op ARF, sepsis	Pseudomonas aeruginosa	28	8	14
P3	M	59	ALL with neutropenic sepsis and ARF	Empiric cover for sepsis	24	5	10
P4	M	63	Ruptured abdominal aortic aneurysm repair with post-op ARF and sepsis	Acinetobacter baumanni	18	5	13
P5	F	70	Pneumonia, ARDS	Klebsiella pneumoniae	24	2	11
**Mean**		**63.4**		**-**	**26.6**	**6**	**11**

^1^ Initial/ICU admission value.

^2^ Creatinine clearance value on day 1 of CVVHDF, prior to commencing CVVHDF therapy. Units = ml/min; estimated using the method of Jelliffe and Jelliffe.

ARF = Acute renal failure.

ALL = Acute lymphocytic leukaemia.

ARDS = Acute Respiratory Distress Syndrome.

Ethical approval was obtained from the Joint Hospitals Ethics Committees (St James Hospital/Adelaide and Meath Hospital Dublin, Incorporating the National Children’s Hospital Ref No. 041007/7704). Approval was obtained from the Irish Medicines Board. Written informed consent to participate and publish (predominantly consent by proxy) was obtained in compliance with Helsinki declaration.

### CVVHDF procedure

A 0.6 m^2^ polyacrilonitrile cylinder haemofilter (Prisma M100, Preset AN69HF, Hospal, Lyon, France) was utilised. Blood was pumped through the membrane at a rate of 200 ml min^-1^. The dialysate fluid passed once across the membrane into the dialysate compartment of the filter at a rate of 1–2 l h^-1^. The ultrafiltration rate and predilution replacement solution infusion rates were both 2 l h^-1^.

### Administration of amikacin

Multiple doses of amikacin were administered to each patient. Each dose of amikacin was infused intravenously over a period of 30 minutes. Blood samples (7 ml) were taken immediately prior to the administration of a subsequent dose (trough) and 30 minutes after the infusion was complete (peak).

Additionally, multiple serum concentrations (minimum of seven; at the end of the infusion, and at 1, 2, 5, 8, 12, 18 hours) in a dosage interval were obtained for at least one dosage interval for each patient.

In the hospital setting of this study, current target peak and trough plasma concentrations using once-daily dosing (extended interval dosing) are 50–60 mg/L and <5 mg/L respectively and these criteria are consistent with literature ranges of approximately 40 - 60 mg/L and <5 mg/L [4,21,22]. It should be noted that the study time period straddled the hospital’s transition from multiple daily dosing (7.5 mg kg^-1^ twice daily, trough <2.5 mg l^-1^, peak not greater than 30 mg l^-1^) to once daily/extended interval dosing.

### Analytical Procedures

Concentrations of amikacin in serum and dialysis effluent fluid were measured using a TDx analyser (Abbott) using a fluorescence polarization immunoassay. An amikacin standard concentration curve was constructed using calibrators (0, 5.0, 10, 20, 30 and 50 mg l^-1^). Blood samples were stored at 4°C prior to prompt analysis.

Amikacin clearance by CVVHDF was investigated for a single dosage interval. The amount of amikacin in each effluent collection during the dosage interval was calculated by multiplying the measured effluent concentration in each effluent sample by the volume of effluent collected over the corresponding time period. Effluent samples were stored at 4°C pending assay.

### Pharmacokinetic analysis

#### Multiple concentration data over a dosage interval – 1 compartment model

Multiple serum concentrations during one dose interval were used to determine k, V_d_, t_1/2_ and total body clearance (TBC). k was calculated through fitting of the concentration time data to a 1-compartment infusion model using WinNonlin pharmacokinetic software version 5.2, (Pharsight Corporation, North Carolina, USA).

#### Multiple concentration data over a dosage interval - 2 compartment model

It was attempted to fit the concentration-time data from multiple samples taken over a dosage interval to a 2-compartment infusion model using WinNonlin version 5.2, (Pharsight Corporation, North Carolina, USA). Fitting multiple serum concentration data to a 2-compartment model facilitated the calculation of TBC, V_1_, k, t_1/2 (el)_, t_1/2α_, t_1/2β_ and Vss. Vss is the estimated volume of distribution at steady state. In this case, t_1/2(el)_ is the elimination half life, comparable to t_1/2_ for the one compartment model. In order to calculate initial estimates, the post-infusion concentration data was fitted to a bi-exponential curve of the form C_pt_=A_1_e^-αt^+B_1_e^-βt^, where C_pt_ is the plasma concentration at time, t. The parameters A_1_ and B_1_ were then transformed to A and B using equation 1, where T is the duration of the infusion, C_i_ = A or B, Y_i_ = A_1_ or B_1_ and λ_i_ = α or β [23].

(1) $$C_{i}=\frac{\lambda_{i}TY_{i}}{e^{\lambda_{i}T}-1}$$

The relevant pharmacokinetic parameters were then calculated using the following standard equations:

(2) $$\frac{A\beta +B\alpha }{A+B}=k_{21}$$

(3) $$k_{10}=\frac{\alpha \beta }{k_{21}}$$

(4) $$V_{1}=\frac{Dose}{A+B}$$

where V_1_ is the volume of the central compartment.

#### Peak and trough values

In order to evaluate serum concentration data which would be available under routine therapeutic drug monitoring conditions, individualized pharmacokinetic parameters were determined from each patient's peak and trough serum concentration data using the method of Sawchuk and Zaske [24]. The half life (t_1/2_), elimination rate constant (k), TBC and volume of distribution (V_d_) were calculated as follows:

(5) $$k=\frac{\left(\ln C_{p\max}-\ln C_{p\min}\right)}{\tau -t^{1}}$$

where τ is the dosage interval and t^1^ is time of the peak sample.

(6) $$t_{\frac{1}{2}}=\frac{0.693}{k}$$

(7) $$V_{d}=\frac{\frac{D}{T}\left(1-e^{-kT}\right)}{k\left(C_{p\max}-\left(C_{p\min^{1}}e^{-kT}\right)\right)}$$

where D is the dose, T is the duration of the infusion and $C_{p\min^{1}}$ is the trough concentration from the previous dose [24]. In the case of the first dose, the term $C_{p\min^{1}}e^{-kT}$ was omitted from Equation 7.

(8) $$TBC=k\times V_{d}$$

## Results

### Patient demographics

Three men and two women treated with amikacin during CVVHDF therapy, ages 57–70 (mean +/− SD: 63.4 +/− 5.6 years) were enrolled in the study. Demographic data and clinical characteristics are given in Table 1. All five patients enrolled in the prospective study had severe renal impairment. Three were anuric throughout treatment (patients 1, 2, 3) and two patients were anuric on commencing CVVHDF but became oliguric during treatment (day 11 and day 10), at which time treatment with amikacin had stopped. The average measured effluent flow rate was 3.5 +/−0.6 l h^-1^.

The mean APACHE II (Acute Physiology and Chronic Health Evaluation II) score was 26.6 +/− 7.8. The mean duration of CVVHDF therapy was 11.0 +/− 2.7 days. Patients 1–4 were diagnosed with sepsis and two patients (1 and 4) had concomitant liver and renal impairment.

### Pharmacokinetic results

In total 68 amikacin concentrations were determined, ranging from 1.8 mg l^-1^ to 68.3 mg l^-1^. All samples were quantifiable.

#### Measuring multiple serum concentrations over a dosage interval and fitting to 1- and 2-compartment models

**Figure 1:**
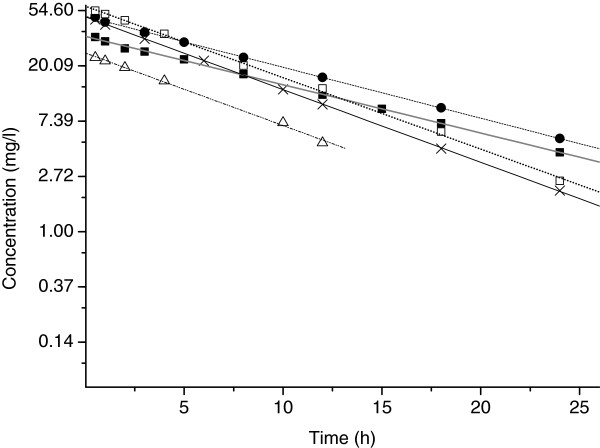
**Semi-log plot of multiple amikacin serum concentrations in a dosage interval over time, and (linear fit) for all patients treated concurrently with amikacin and CVVHDF.** Key: ■ patient 1 ● patient 2 х patient 3 ∆ patient 4 □ patient 5.

The multiple amikacin serum concentrations sampled in a dosage interval for each of the five patients are presented graphically in Figure 1. This post infusion data was analysed in terms of the 1- and, where possible, 2-compartment open models, and the parameters obtained are summarized in Tables 2 and 3 respectively.

**Table 2:** Individual patient estimates of amikacin pharmacokinetic parameters during treatment with CVVHDF, obtained from multiple amikacin serum concentrations in a dosage interval fitted to a one-compartment model

**ID**	**k (h**^**-1**^**)**	**CV%_k**	**t**_**1/2**_**(h)**	**V**_**d**_**(l)**	**TBC (l h**^**-1**^**)**
P1B	0.088	2.8	7.91	28.47	2.49
P2C	0.094	2.31	7.37	31.97	3.01
P3C	0.130	1.27	5.33	32.48	4.22
P4B	0.141	5.99	4.91	29.45	4.15
P5C	0.126	1.98	5.49	27.88	3.51
**Mean +/−**	**0.116 +/−**		**6.21**	**30.05**	**3.48**
**s.d.**	**0.024**		**+/−**	**+/−**	**+/−**
			**1.34**	**2.07**	**0.74**
**Median**	**0.126**		**5.5**	**29.45**	**3.51**

t_1/2:_ half life. V_d_: volume of distribution. TBC: Total body clearance. k: elimination rate constant. CV%: coefficient of variation. PX (1–5) patients 1–5.

**Table 3:** Individual patient estimates of amikacin pharmacokinetic parameters during CVVHDF therapy assuming a two compartment model

**ID**	**k (h**^**-1**^**)**	**V**_**1**_**(l)**	**TBC (l h**^**-1**^**)**	**T**_**1/2α**_**(h)**	**T**_**1/2β**_**(h)**	**T**_**1/2el**_**(h)**	**V**_**ss**_**(l)**
P1B	0.093	25.0	2.32	0.25	8.18	7.45	27.33
P2C	0.131	21.95	2.88	0.035	7.61	5.29	31.5
P3C	0.172	24.31	4.19	0.028	5.29	4.02	31.94

t_1/2α_: half life of the alpha phase. t_1/2β_: half life of the beta phase. t_1/2el_: elimination half life. V_ss_: volume of distribution at steady state. V_1_: volume of distribution of the central compartment. TBC: Total body clearance. k: elimination rate constant. CV%: coefficient of variation. PX (1–5) patients 1–5.

Estimates of the pharmacokinetic parameters, k, t_1/2_, TBC and V_d_, were initially obtained for each patient on the basis of a 1-compartment model. The results are presented in Table 2.

The same amikacin serum concentration time data, fitted to a 2-compartment model gave estimates of the pharmacokinetic parameters, k, t_1/2(el)_, t_1/2α_, t_1/2β_, V_1_, TBC and V_ss_ shown in Table 3. It was not possible to reasonably fit the data for patients 4 and 5 to a 2-compartment model. The best fit to a 2-compartment model was with the data from patient 1 where a short but clear distribution phase was evident. Nevertheless, the highest CV% values were associated with the estimates for k21, t_1/2α_ and V_2_, illustrating the variability surrounding the fitting of data to the distribution phase, when it is not very distinct and only captured by 1–2 points. Although the data was fitted to a 2-compartment model for patients 2 and 3, the standard residual and CV% values were much higher for all parameters, and more so those associated with the distribution phase and the second compartment. Overall the relevant pharmacokinetic parameters were similar in Tables 2,3, when considering the values from the same patient. Therefore, the current data suggest that, although it was possible to fit the data to a 2-compartment model in some cases, parameter estimates were similar to those obtained with a 1-compartment model. Most importantly, as can be seen from Figure 1, there was little or no distribution phase evident from the concentration-time profiles, further supporting the use of a 1-compartment model for pharmacokinetic analysis of this data. A short distribution phase is anticipated with aminoglycoside antibiotics, which is generally expected to be complete by 30 minutes after the end of the 30 minute infusion [4]. Therefore, in the case of these patients the distribution phase was evidently very rapid and of a small magnitude in comparison with the magnitude and time scale of the rest of the concentration-time profile.

#### Estimates of pharmacokinetic parameters and dosage recommendations using peak and trough data only

Individual patient estimates of amikacin pharmacokinetic parameters during treatment with CVVHDF were obtained from amikacin serum concentration data. Estimates of t_1/2_, k, V_d_, and TBC obtained from peak and trough values are presented for each patient in Table 4.

**Table 4:** Peak and trough concentration data and estimates of amikacin pharmacokinetic parameters

**Patient profile**	**Dose**	**Dosage interval**	**Cpmax**	**Cpmin**	**t**_**1/2**_	**k**	**TBC**	**V**_**d**_
	**(mg)**	**(h)**	**(mg l**^**-1**^**)**	**(mg l**^**-1**^**)**	**(h)**	**(h**^**-1**^**)**	**(l h**^**-1**^**)**	**(l)**
P1A	900	24	31.3	3	6.80	0.102	2.858	28.03
P1B	900	24	33.8	4	7.63	0.091	2.583	28.44
P1C	1100	24	35.7	6.2	9.10	0.076	2.579	33.89
**Mean**					**7.84**	**0.090**	**2.674**	**30.12**
P2A	300	19	7.6	1.8	8.66	0.080	3.096	38.69
P2B	1500	24	50.2	2.8	5.52	0.125	3.761	29.97
P2C	1500	24	48.5	5.4	7.42	0.093	2.987	31.98
P2D	1500	29	49.4	5	8.47	0.082	2.719	33.24
P2E	1500	32	55.9	6.1	9.70	0.071	2.062	28.85
P2F	1500	29	53.8	7	9.51	0.073	2.239	30.74
**Mean**					**8.21**	**0.088**	**2.811**	**32.25**
CVVHDF Stopped P2G	1500	53.5	68.3	16.1	25.18	0.028	0.668	24.26
P3A	1500	24	44.9	2.8	5.74	0.121	3.911	32.42
P3B	1500	24	42.4	1.9	5.13	0.135	4.923	36.46
P3C	1500	24	46.5	2.1	5.26	0.132	4.278	32.46
**Mean**					**5.38**	**0.129**	**4.371**	**33.78**
P4A	600	12	16.2	4	5.45	0.127	4.563	35.88
P4B	600	12	23.4	5	5.16	0.134	3.961	29.52
**Mean**					**5.31**	**0.131**	**4.262**	**32.70**
P5A	1500	24	48.2	2.1	5.09	0.136	4.099	30.08
P5B	1500	25	52.4	2.4	5.39	0.128	3.701	28.81
P5C	1500	28	54.4	2.5	5.29	0.131	3.649	27.84
P5D	1500	28	55.8	2.5	6.03	0.115	3.137	27.28
**Mean**					**5.45**	**0.128**	**3.646**	**28.50**
Overall mean*					**6.74**	**0.109**	**3.39**	**31.4**
**Standard deviation**					**1.69**	**0.025**	**0.82**	**3.27**
**Median**					**5.88**	**0.118**	**3.39**	**30.4**

* Excluding profile P2G.

Cpmax: maximum serum concentration Cpmin: minimum serum concentration.

t_1/2:_ half life. V_d_: volume of distribution. TBC: Total body clearance. k: elimination rate constant.

PX (1–5): patients 1–5. PXA-PXE: first (A) to fifth (E) dosing interval during which serum concentration data was collected for patient PX.

Initial doses in patients 1, 2 and 4 were commenced prior to pharmacokinetic recommendations being available, according to the then current dosing practice on the ICU (pre-dating the change in dosing strategy). Subsequently pharmacokinetic parameters calculated for patients 1 and 2 were similar, and a dosing regime of 1500 mg at intervals of 31–32 hours was recommended. The initial results from these two patients illustrate the confusion which can surround “once daily” vs. “extended interval” dosing. The initial dose (900 mg) in patient 1 was subsequently increased to 1100 mg every 24 hours. Possibly due to the high V_d_ observed in this patient, this dose was not adequate to achieve recommended peak levels, however the interval of 24 hours contributed to a degree of accumulation. This dosing schedule did not achieve target C_pmax_ concentrations in this patient during CVVHDF therapy.

Subsequently, in patient 2, the recommended dose of 1500 mg was prescribed, achieving recommended peak concentrations. However the interval was not increased immediately and accumulation was noted. The interval was increased on day 5 and the rate of accumulation decreased.

**Figure 2:**
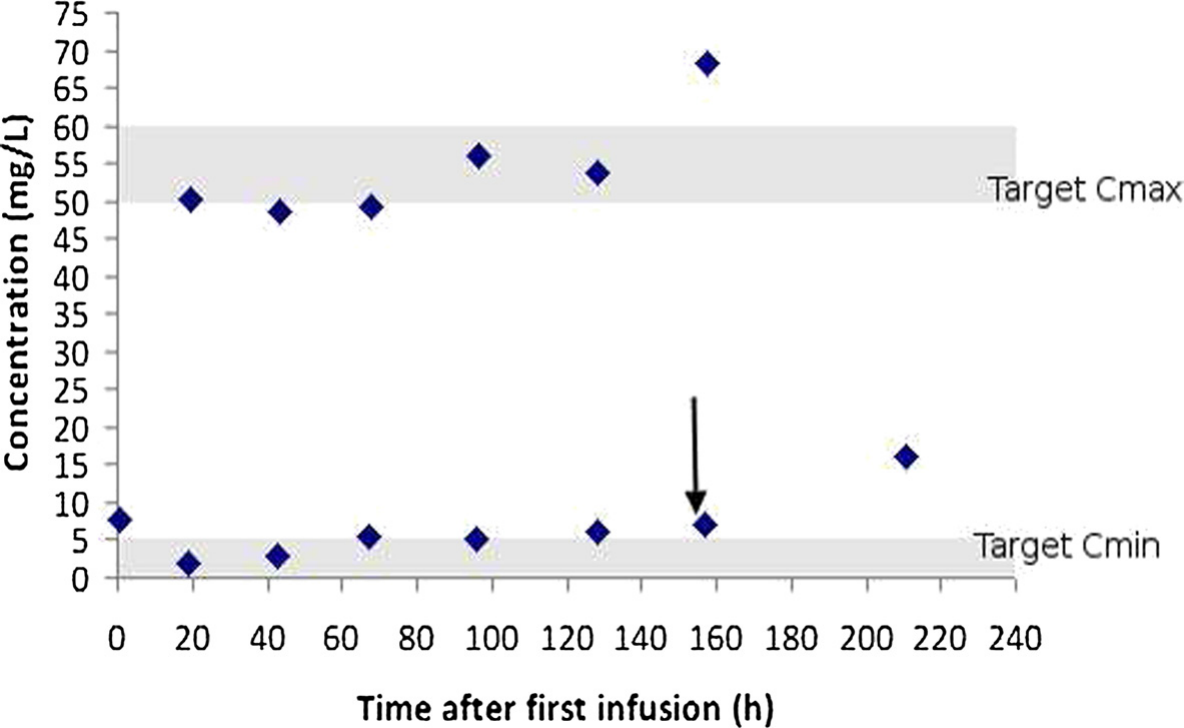
**Amikacin serum concentration-time data for Patient P2.** The arrow indicates the point at which CVVHDF was ceased. The shaded areas indicate the target therapeutic peak and trough ranges.

In the case of patient 2, stopping CVVHDF therapy resulted in a three-fold increase in the amikacin half-life. Figure 2 shows amikacin serum concentrations over time for Patient 2. The arrow indicates the point at which CVVHDF therapy was stopped and a subsequent increase in serum concentrations was observed. Despite the significant increase in interval between peak and trough sampling (53.5 h), the need for a further extension in dosage interval and decrease in dose due to the decrease in clearance after CVVHDF was discontinued is evident from the high trough level.

For patient 3, a 1500 mg dose achieved adequate C_pmax_ concentrations, while the C_pmin_ concentrations were also below the target threshold with a dosage interval of 24 hours. The mean clearance estimate, 4.4 +/− 0.51 l h^-1^, was high and this value was close to the mean observed effluent flow rate during CVVHDF (4.1 l h^-1^).

As mentioned in the methods section, this study straddled the period of transition from multiple daily dosing to extended interval dosing of aminoglycosides. In the case of patient 4, Acetinobacter baumanni was isolated from sputum samples and amikacin therapy was initiated. Instead of administering a high dose at extended interval, 600mg amikacin was administered twice daily. The C_pmax_ concentration achieved by the 600 mg dose was 16.2 mg/L and the C_pmin_ concentration was 4 mg l^-1^, which is above the recommended trough level. If the patient had continued on amikacin following the second dose, an extended interval should have been considered due to the decrease in clearance and higher than recommended trough values.

**Figure 3:**
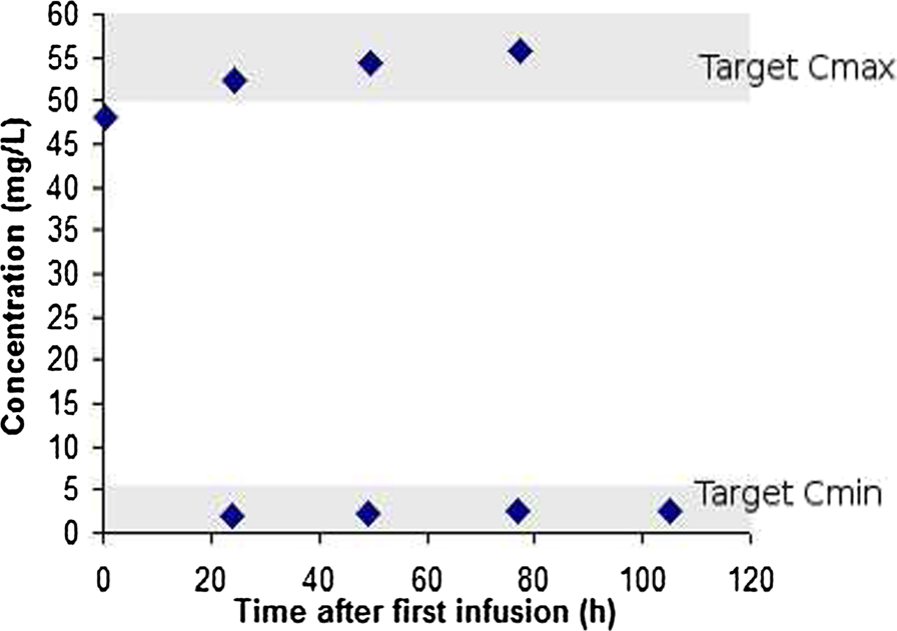
**Amikacin serum concentration time data for Patient P5.** The shaded areas indicate the target therapeutic peak and trough ranges.

For patient 5, a dosing schedule of 1500 mg once daily achieved effective C_pmax_ concentrations, based on a target C_pmax_/MIC ratio of 10. The MIC for the sensitive microorganism was 5ug ml^-1^ amikacin. For profiles C and D, the dosage interval was extended somewhat to avoid excessively high C_pmin_ concentrations. Serum concentration-time data for this patient, illustrating the dosing regime accomplishing target concentrations, is depicted in Figure 3.

The estimates of pharmacokinetic parameters from the 1-compartment model, obtained from the multiple amikacin serum concentration versus time data, can also be compared with pharmacokinetic parameters estimates (TBC, V_d_, k) obtained from typical TDM data (Sawchuk and Zaske method) from the same patient profiles. It is clear that values calculated using both methods for each patient are similar (Tables 2 and 4). The data from this study therefore supports the usefulness of the Sawchuk and Zaske method to obtain appropriate amikacin PK parameters for patients on CVVHDF. These parameters are suitable for dosage regimen calculation/adjustment, thereby supporting a practical approach of individualising amikacin dosing based on routinely available troughs and peaks in patients on CVVHDF.

#### Amikacin Clearance due to CVVHDF

Details of amikacin and creatinine clearances obtained during CVVHDF, together with the CVVHDF conditions employed are given in Table 5. The mean clearance of amikacin by CVVHDF was 2.86 +/− 0.41 l h^-1^, ~89% of the mean total body clearance for these patient profiles. The sieving coefficient for amikacin was 0.83 +/− 0.05, which was consistent with that previously reported in the literature (0.93 +/− 0.16), although different filters and CRRT conditions were in use [13]. The observed sieving coefficient was similar to the unbound fraction of amikacin, assuming a fraction unbound of 0.8 based on the fact that 20% or less of amikacin is bound to serum protein [3]. The amikacin clearance due to CVVHDF estimated using the sieving coefficient, 2.93 l h^-1^, was similar to the actual measured clearance (2.86 l h^-1^). Creatinine clearance (2.74 +/− 0.42 l h^-1^) by the filter was similar to amikacin clearance. The sieving coefficient for creatinine was 0.80 +/− 0.1. This value was very close to the sieving coefficient for amikacin, but was slightly lower and more variable.

**Table 5:** Clearance of amikacin and creatinine by CVVHDF and summary of CVVHDF conditions

**Patient profile**	**Cl**_**CVVHDF**_**(l h**^**-1**^**)**	**F**_**CVVHDF**_	**CrCl (l h**^**-1**^**)**	**Actual effluent flow rate (l h**^**-1**^**)**	**Number. of filters ^**	Age of filter* (h)
P1C	2.53	0.98	2.19	3.16	1	16
P2D	2.55	0.94	2.70	2.91	1	27
P3A	3.40	0.87	3.20	4.10	1	1
P5B	2.97	0.80	2.86	3.95	1	26
**Mean**	**2.86**	**0.90**	**2.74**	**3.53**	**1**	**-**
**+/−**	**+/−**	**+/−**	**+/−**	**+/−**		
**s.d.**	**0.412**	**0.08**	**0.42**	**0.584**		

*At start of dosage interval.

^ During dosage interval.

PX (1–5) patients 1–5. Cl_CVVHDF_: clearance via CVVHDF F_CVVHDF_: Fraction of total body clearance via CVVHDF. CrCl: Creatinine clearance.

## Discussion

CVVHDF was observed to increase amikacin clearance, with a mean TBC value of 3.39 l h^-1^. The results demonstrate that CVVHDF is capable of significant amikacin clearance, accounting for most of the measured TBC in all patients. As such, the CVVHDF status of a patient, i.e. whether CVVHDF is commenced, temporarily interrupted or discontinued, is of primary importance when estimating the dose regimen.

The mean half-life during CVVHDF therapy was 6.74 hours, and the median half-life estimate of 5.88 h comparing well with that of 6.5 h reported by Taccone et al. [17] Additionally, the mean elimination rate constant was 0.109 h^-1^ indicating that elimination is ~ one third that observed in subjects with normal renal function. In patients with normal renal function, the half-life is 2–3 hours but in anephric patients, the half-life increases to 30–60 hours [4]. A wide range of values for the V_d_ of aminoglycosides (0.1 to 0.5 l kg^-1^ ~ 7–35 l)) has been reported and the observed mean value from the current study (31.4 +/− 3.27 l) lies at the higher end of this range (based on Ideal Body Weight (IBW)). Assuming a 70 kg patient weight, this corresponds to a value of 0.45 l kg^-1^, which is comparable to the estimate of V_ss_ of 0.5 l kg^-1^ determined by Taccone et al. [17]. Interestingly, the value for V_1_ reported in that study was lower (median 0.29 l kg^-1^) than estimated in the current work; however it ranged from 0.21-0.62 l kg.

A large volume of distribution in critically ill patients is not unexpected as sepsis [10], total parenteral nutrition and factors associated with critical illness such as aggressive fluid therapy and hypoalbuminemia have been associated with an increased volume of distribution for aminoglycoside antibiotics [25]. As a result of this increased volume of distribution and the fact that aminoglycosides demonstrate concentration dependent killing, higher loading doses may be required to obtain clinically relevant peak concentration values, as is occurring in practice [17].

‘Once-daily’ aminoglycoside dosing is more correctly described as extended-interval dosing and extension of the dosage far beyond 24 hours will be required in some patients with renal dysfunction. The importance of acknowledging this concept, rather than attempting to maintain a rigid ‘once-daily’ dosage interval, is illustrated in the cases of patients 1 and 2. In the treatment of the first patient, where strict ‘once-daily’ dosing was applied, lower doses at 24-hour intervals failed to achieve target peak concentrations. The high V_d_ combined with a lower dose in patient 1 contributed to the peak concentration failing to reach the recommended target value. The risk of accumulation following higher doses to achieve appropriate peak concentrations has also been highlighted by Taccone et al. [17], and evidence is provided in the current work to support this concern, where accumulation was evident in patients 1 and 2. However, in contrast to patient 1, in the case of patient 2, use of the recommended dose and extension of the dosage interval beyond 24 hours, allowed target peak serum concentrations to be achieved, while limiting accumulation. Estimates of amikacin pharmacokinetic parameters were similar for both patients and the dosage recommendation for patient 2 was 1500 mg every 32 hours. It is imperative that clinicians be aware of the significant impact of CVVHDF on amikacin clearance and thus the effect of stopping, interrupting or changing dialysis modality on amikacin serum concentrations. The observed effect of stopping CVVHDF was evident in patient 2 (Figure 2).

This study supports the approach of using routinely measured (both peak and trough) amikacin serum levels in estimating pharmacokinetic parameters and thus guiding dosage regimens. For each patient the values of the pharmacokinetic parameters calculated from peak and trough data were similar to those calculated from multiple serum concentration data within a dosing interval fitted to a 1-compartment model. As obtaining multiple serum concentrations within a dosing interval would not be practical in the routine clinical setting, a standard TDM approach of using peak and trough data only is shown to be suitable for obtaining ongoing meaningful individual pharmacokinetic data. Use of regularly obtained peak and trough data to assess individual pharmacokinetic parameters is likely to be particularly valuable in the context of critically ill patients receiving CRRT. This is due to the complex interaction between the patient, drug factors and CRRT factors in influencing drug pharmacokinetics.

Careful consideration should be given to the importance of the timing of the peak and trough sampling. Although several of the profiles in the current work had no discernible distribution phase, the peak sample should be taken approximately 30 minutes after the end of the infusion [4]. It has been suggested that in some cases a longer distribution phase may be present [4]. In any case, consideration should always be given to the timing of the peak sample data used to generate the target peak range used. Furthermore, trough samples should be taken 2–4 half lives after the peak sample [4]. In addition, caution must be applied in the interpretation of drug serum levels when interruptions in CVVHDF therapy have occurred, for example due to filter clotting or medical interventions.

It has been suggested that the reduced amikacin half life observed in the study by Robert et al. [12], of patients receiving CVVH, compared to that reported by Armendariz et al. [13] arose because of the higher haemofiltration rate contributing to the higher observed clearance of amikacin. Furthermore, the longer observed half life during CVVH compared with CVVHDF is consistent with an increased dialysis efficiency from CVVHDF in comparison with CVVH [15]. Therefore it would be anticipated that shorter half lives would be observed in patients undergoing CVVHDF in comparison with those observed in patients undergoing CVVH, as demonstrated by the current study. These differences in observed half lives underline the relevance of continuous renal replacement therapy mode employed and conditions used (e.g. flow rate) when considering potential for drug clearance during dialysis. In addition to the study by Taccone et al. [17], at present, the authors are aware of only one other prospective analysis of amikacin pharmacokinetics in patients undergoing CVVHDF. That study reported a mean half life of 11.3 h (+/− 1.5) [16] which is longer than that determined in the current study. This is likely explained by higher blood flow rates (100 ml min^-1^ vs. 200 ml min^-1^ in the current study), dialysate flow rates and pre-dilution replacement flow rates (1 l h^-1^ vs lh^-1^ to 2 lh^-1^ in the current study). Although both the current study and that by Moon et al. [16] found evidence of significant amikacin clearance via CVVHDF, the differences in calculated pharmacokinetic parameters further illustrate the effect of CVVHDF conditions employed on amikacin clearance. On the other hand, no correlation was found by Taccone et al. between CVVHDF conditions employed and calculated clearance or trough levels within the range of conditions employed. The observed clearance values [17] were thought to be affected by residual renal function and/or potential filter absorbance. Due to this lack of clarity, a more comprehensive study of CVVHDF parameters employed and amikacin levels is warranted to ascertain the effect of CVVHDF conditions employed on amikacin clearance.

It has been recommended recently that more aggressive dosing practices need to be employed for patients undergoing dialysis who are treated with aminoglycosides, as poorer outcomes were observed retrospectively among dialysis patients receiving aminoglycosides, with one of the risk factors for mortality being lower peak concentrations relative to MIC [26]. Recently CVVHDF was employed to enable use of high amikacin doses to achieve appropriate peak levels while avoiding nephrotoxicity [19]. This approach should contribute to prevention of treatment failure and minimisation of the emergence of resistance. This illustrates that there is a growing acceptance of both the high peak levels of aminoglycosides required in many cases along with the potential for significant clearance via CVVHDF. However, to date data have been lacking to support initial dosage regimen estimates and the expected range of individual pharmacokinetic parameters for amikacin in critically ill patients on CVVHDF.

### Limitations of the current study

The limited patient number (5) in this study, together with their heterogeneity, limits in-depth statistical analysis of the data. The results are presented as the ranges and absolute values which might be expected from 5 critically ill patients undergoing CVVHDF.

## Conclusions

CVVHDF contributes significantly to total clearance of amikacin. The use of pharmacokinetic parameter estimates obtained from two appropriately timed steady state serum-drug concentrations (peak and trough) can be used to guide individualised dosing of critically ill patients treated with CVVHDF. This is considered a useful monitoring strategy particularly in avoiding the risk of underdosing. The potential for underdosing coupled with the notable decrease in clearance when CVVHDF is discontinued indicates that individualised dosing of patients treated with CVVHDF using estimates of pharmacokinetic parameters is required.

## Abbreviations

Cl: Clearance; C_pmax_: Maximum plasma concentration; C_pmin_: Minimum plasma concentration; CrCl: Creatinine clearance; CV%: Coefficient of variation; CVVH: Continuous venovenous haemofiltration; CVVHDF: Continuous veno-venous haemodiafiltration; ESBL: Extended spectrum beta lactamases; F_CVVHDF_: Cl via CVVHDF as fraction of TBC; ICU: Intensive care unit; k: Elimination rate constant; MIC: Minimum inhibitory concentration; T: Duration of infusion; t_1/2_: Half life; TBC: Total body clearance; TDM: Therapeutic drug monitoring; V_1_: Volume of central compartment; V_2_: Volume of peripheral compartment; V_d_: Volume of distribution; Vss: Volume of distribution at steady state.

## Competing interests

The authors declare that they have no competing interests.

## Authors’ contributions

OC, CG, and MD initiated and supervised the study from its inception. EC co-ordinated sample and consent acquisition. DD completed the pharmacokinetic analysis and modelling. OC authored the first draft, with review and additions by DD, MD and CG. OC, CG, MD, EC and DD read and approved the final manuscript.

## Pre-publication history

The pre-publication history for this paper can be accessed here:

http://www.biomedcentral.com/2050-6511/13/14/prepub
